# Early Vitrectomy with Silicone Oil Tamponade in the Management of Postoperative Endophthalmitis

**DOI:** 10.3390/jcm12155097

**Published:** 2023-08-03

**Authors:** Constance Weber, Isabel Stasik, Philipp Herrmann, Steffen Schmitz-Valckenberg, Frank G. Holz, Raffael Liegl

**Affiliations:** 1 Department of Ophthalmology, University of Bonn, 53127 Bonn, Germany; constance.weber@ukbonn.de (C.W.); isabel.stasik@ukbonn.de (I.S.); philipp.herrmann@ukbonn.de (P.H.); steffen.schmitz-valckenberg@ukbonn.de (S.S.-V.); frank.holz@ukbonn.de (F.G.H.); 2 John A. Moran Eye Center, Department of Ophthalmology & Visual Sciences, University of Utah, Salt Lake City, UT 84132, USA

**Keywords:** endophthalmitis, vitrectomy, silicone oil

## Abstract

**Background:** Early vitrectomy for postsurgical endophthalmitis may improve visual acuity outcomes. Silicone oil as a tamponade has some potential benefits in the management of endophthalmitis. This study aims to evaluate the use of a silicone oil tamponade in the surgical management of endophthalmitis. **Material and Methods:** All patients with a pars plana vitrectomy with silicone oil tamponade for postsurgical endophthalmitis at the Department of Ophthalmology, University of Bonn, Germany, between 2017 and 2021 were retrospectively reviewed. We included all preoperative data, including BCVA at diagnosis, clinical findings, and symptoms. For every follow-up visit, we looked at BCVA and complications. **Results:** In total, 82 patients were included in this study. The mean follow-up was 13.1 months (range 1–58 months). An intravitreal injection was the cause in 42 patients (51.2%) and cataract surgery in 29 patients (35.4%). The mean interval between the causing event and the date of onset was 8.8 days (range, 1–59 days). The most prevalent pathogen was Staphylococcus epidermidis in 16 patients (19.5%). In 47 patients (57.3%), no pathogen was found. The initial best-corrected visual acuity was 2.1 logMAR and improved significantly to 1.0 logMAR after six months (*p* < 0.001) and 1.1 logMAR after 1 year (*p* < 0.001). In a multivariate analysis, a low BCVA at diagnosis (*p* = 0.041) was a significant predictor for poor visual acuity outcomes. A total of 17 patients (20.1%) developed postoperative complications. Five patients (6.1%) needed an anterior chamber washout with repeated injections of antibiotics. Two patients (2.4%) had persistent fibrin and were treated with YAG-laser treatment. Three patients (6.7%) developed a retinal detachment. Two patients (2.4%) had persistent corneal decompensation with endothelial cell loss and received perforating keratoplasty. We performed a matched-pair analysis (*n* = 30, each group *n* = 15) to compare a silicone oil tamponade with BSS at the end of surgery. The visual acuity outcome showed no significant differences (BCVA after one year: 1.17 logMAR in eyes with silicone oil and 0.90 logMAR in eyes with BSS; *p* = 0.684). **Conclusions:** In our study, a vitrectomy with silicone oil tamponade in the surgical management of postoperative endophthalmitis led to a significant improvement in visual acuity and had a low complication rate. Low BCVA at diagnosis was significantly associated with poor visual acuity outcomes. A comparison of silicone oil and BSS at the end of surgery showed similar results.

## 1. Introduction

Endophthalmitis is the most feared complication following intraocular surgery. This severe ocular infection most commonly occurs in patients who have previously either received cataract surgery or intravitreal injection (IVI). It can also occur due to an endogenous cause. Although rare, endophthalmitis can lead to severe vision loss. After cataract surgery, the reported rate of endophthalmitis ranges between 0.04 and 0.2% [1]. The rate after IVI is similar, ranging from 0.008 to 0.092% [2,3,4]. Similar rates have been reported for vitrectomy, with an incidence ranging from 0.03 to 0.11% [5] and for penetrating keratoplasty, with rates from 0.14 to 0.38% [6].

Because time to treatment is critical, it is particularly important to diagnose patients with endophthalmitis as soon as possible so that prompt treatment can be initiated to prevent or at least mitigate vision impairment.

In 1995, the Endophthalmitis Vitrectomy Study (EVS) was conducted to investigate and compare treatment options in the management of endophthalmitis. To date, this study is still the only randomized controlled trial that has addressed this important issue [7]. It investigated the role of immediate vitrectomy for patients with postoperative endophthalmitis and compared it to patients receiving a vitreous tap or biopsy (TAP) in addition to intravitreal (IV) antibiotics. In addition, the role of systemic antibiotic treatment was evaluated. The EVS concluded that patients who presented with a visual acuity of hand motion or better did not benefit from immediate vitrectomy in comparison to patients with TAP and IV antibiotics, whereas patients with a visual acuity of only light perception did. Furthermore, it was reported that systemic antibiotic treatment reduced toxic effects and the length of hospitalization. 

Given the significant improvements in surgical equipment and instrumentation, it is unclear to what extent these results can still be translated into clinical practice today. Recent retrospective studies suggest that treatment algorithms should be adapted to current conditions.

Still, the mainstay of treatment is the intravitreal administration of antibiotics. Whether systemic administration of antibiotics has a positive effect on clinical outcomes is still unclear [8].

Studies point out that vitrectomy may improve functional outcomes in endophthalmitis [9]. However, it remains unclear which patients benefit from vitrectomy and at what point the procedure should be performed. Most recent studies show that patients’ vision recovers better when vitrectomy is performed as early as possible after diagnosis [10,11,12].

Few studies have examined different tamponade options during vitrectomy. Silicone oil showed antimicrobial activity in in vitro experiments and might therefore be beneficial as a tamponade in endophthalmitis patients [13,14]. It showed activity against pathogens that cause endophthalmitis, such as *Staphylococcus* (*S.*) *epidermidis* or *S. aureus* [15]. It was also shown that silicone oil as a tamponade has a positive effect when a retinal detachment is present in order to prevent further damage [16]. Furthermore, it can be beneficial in eyes with severe endophthalmitis in order to prevent retinal breaks [14,17] or in cases of post-traumatic endophthalmitis [18]. 

Performing a vitrectomy seems essential to restoring vision, especially in eyes with severe endophthalmitis. Still, there are no recommendations on which tamponade to use when performing a vitrectomy in cases of endophthalmitis. This study was performed to investigate the outcome of pars plana vitrectomy with silicone oil tamponade in the management of postoperative endophthalmitis.

## 2. Materials and Methods

### 2.1. Patients

Medical records of all patients who were treated with a pars plana vitrectomy and silicone oil tamponade due to the development of endophthalmitis at the Department of Ophthalmology, University of Bonn, Germany, between January 2017 and December 2021 were retrospectively reviewed. Only patients with exogenous endophthalmitis were included for further analysis. Symptoms and clinical signs for the diagnosis of endophthalmitis included visual deterioration, pain, conjunctival injection, hypopyon, non-visibility of retinal vessels, and an increased reflectivity of the vitreous in ophthalmic sonography. The diagnosis was based upon the retinal surgeon’s discretion, and not all criteria had to be present in one patient.

All patients received a full ophthalmologic investigation upon presentation, including the assessment of the best-corrected visual acuity (BCVA) measured in Snellen decimal visual acuity and converted to logMAR (hand movement equal to 2.3 logMAR, counting fingers equal to 1.9 logMAR) for statistical analysis, measurement of intraocular pressure, slit lamp biomicroscopy, funduscopy, and ultrasound. All patients received an immediate (within 5 h after presentation) pars plana vitrectomy with vitreous biopsy and anterior chamber tap and application of intracameral and intravitreal antibiotics (vancomycin 1 mg, ceftazidime 2.27 mg, and clindamycin 1 mg) with a silicone oil tamponade. All patients received silicone oil (2000 centistokes). No patient received additional intravitreal dexamethasone. Every patient was treated with systemic antibiotics (IV ceftazidime 2 g 3/d and vancomycin 1 g 2/d; in cases of renal insufficiency, daptomycin 6 mg/kg body weight and ceftazidime 2 g 3/d). The documented data included preoperative clinical features, symptoms at presentation, details of the surgical procedure, systemic treatment, and detailed follow-up regarding complications and BCVA. Further, we identified suitable patients with BSS at the end of surgery in order to perform a matched-pair analysis for a comparison of silicone oil tamponade and BSS.

All analyses were conducted on a deidentified dataset. A waiver by the local ethics committee was granted due to the retrospective character of this study. The study protocol conforms to the ethical guidelines of the 2000 Declaration of Helsinki, as reflected in its a priori approval by the institution’s human research committee. 

### 2.2. Statistical Analyses

Statistical analysis was performed with SPSS Statistics version 27.0.0 (IBM Corporation, New York, NY, USA). We performed a descriptive analysis. For quantitative variables (age; interval between causing event and onset of endophthalmitis; follow-up time; BCVA at diagnosis, 6–12 weeks after surgery, 6 months after surgery, and 1 year after surgery), the mean, range, and standard deviation (SD) before surgery and at different time points after surgery were evaluated. For categorical variables, the total number of patients and their percentage were calculated. We performed matched-pair analysis to compare patients with silicone oil tamponade and BSS at the end of surgery. The normal distribution was evaluated with the Kolmogorov–Smirnov Test. The Pearson’s chi-square test was used to compare the distributions of the nominal- or ordinal-scaled variables. The *t*-test was used for normal distributions, and the Mann–Whitney U-test was used for non-normal distributions in order to compare independent groups. 

A paired sample test was used to compare BCVA at different follow-up time points. Correlation was tested with the Spearman–Rho test. A multivariate regression analysis was performed to identify factors affecting final visual acuity. Variables that were included in this model were: age, gender, visual deterioration, pain, hypopyon, conjunctival injection, visibility of retinal vessels, increased reflectivity of vitreous on B-scan, BCVA at diagnosis, and pathogens for the cause of endophthalmitis. All tests were performed two-sided, and we considered *p* values < 0.05 to be statistically significant.

## 3. Results

A total of 82 eyes from 82 patients who presented to the Department of Ophthalmology, University of Bonn, Germany, and were treated with a pars plana vitrectomy with silicone oil tamponade due to exogeneous endophthalmitis were included in this study. The mean age was 76 years (range, 49–96 y, SD 10.5). A total of 39 women (47.6%) and 43 men (52.4%) were included in the study. The mean time until removal of silicone oil was 3.7 months (1.2–16.8 months, SD: 0.26 months). In four patients, the silicone oil was not removed. The characteristics of our patient cohort are described in Table 1. 

The mean follow-up period was 13.1 months (range 1–58 m). Follow-up data were available for 77 patients at 6–12 weeks, 74 patients at 6 months and 65 patients at 1 year after vitrectomy.

A total of 25 patients (30.5%) had no known systemic diseases. A total of 29 patients (29%) suffered from arterial hypertension, and 17 patients (17%) suffered from diabetes mellitus. 

No concomitant ocular diseases were present in 26 patients (31.7%). However, 32 patients (39.1%) suffered from age-related macular degeneration. Other ocular comorbidities included diabetic retinopathy and diabetic macular edema, open-angle glaucoma, and macular edema due to retinal vein occlusion.

Most patients in our cohort developed endophthalmitis following IVI or cataract surgery. An intravitreal injection was the cause in 42 patients (51.2%) and cataract surgery in 29 patients (35.4%). Other causes for the development of endophthalmitis were vitrectomy (8 patients, 9.7%), glaucoma surgery (1 patient, 1.2%), change of an intraocular lens implant (1 patient, 1.2%), and combined vitrectomy and cataract surgery (1 patient, 1.2%). A total of 78 patients (95.1%) had an acute onset of endophthalmitis within 21 days after the triggering event. The mean interval between the triggering event and the date of onset was 8.8 days (range, 1–59 d). The mean initial BCVA at the time of diagnosis was 2.1 logMAR (range, 0.4–3.0, SD: 0.57), equitable between counting fingers and hand movement. Most patients were pseudophakic at the time of initial presentation (59 patients, 72.0%).

A combined cataract surgery was performed on 11 patients (13.4%). The most prevalent pathogen was *S. epidermidis* in 16 patients (19.5%). In 47 patients (57.3%), no pathogen was found.

The development of BCVA was evaluated at 6–12 weeks (follow-up visit 1), 6 months (follow-up visit 2), and 1 year (follow-up visit 3) after the initial treatment. At 6–12 weeks after surgery, the majority of eyes still had a silicone oil tamponade; there were five patients that had already received a silicone oil removal within six weeks. At follow-up visit one, the mean BCVA was significantly improved to 1.1 logMAR (range, 0.0–3.0 logMAR, SD: 0.69; *p* < 0.001). A total of 68 patients (88.3%) showed an improvement in visual acuity, 4 patients (5.2%) showed a reduction, and 5 patients (6.5%) had no change. 

The mean BCVA at 6 months post vitrectomy presented with 1.03 logMAR (range, 0.1–2.7 logMAR, SD: 0.65; *p* < 0.001). A total of 67 patients (90.1%) showed an improvement in visual acuity. These results were similar to those at the previous time point. The mean BCVA at 1 year post-vitrectomy showed a significant improvement to 1.12 logMAR (range, 0.1–3.0 logMAR, SD 0.8; *p* < 0.001), with 57 patients (87.7%) having an overall improvement in BCVA. The mean difference between the BCVA at diagnosis and the BCVA after one year was 0.91 logMAR (−0.7 logMAR–2.1 logMAR, SD: 0.8).

### 3.1. Complications after Vitrectomy with Silicone Oil Tamponade

Overall, 17 patients (20.1%) developed complications. There were no intraoperative complications. During the first postoperative week, five patients (6.1%) needed an anterior chamber washout with an injection of antibiotics, and one patient (1.2%) developed choroidal folds due to hypotony that regressed without further interventions. During the first three months following surgery, two patients (2.4%) had persistent fibrin in the anterior chamber and received YAG-laser treatment. One patient (1.2%) had self-limiting corneal decompensation.

During the remaining follow-up period, three patients (6.7%) developed a retinal detachment and needed vitrectomy. Two patients (2.4%) had recurrences of intraocular inflammation following silicone oil removal. Two patients (2.4%) had persistent corneal decompensation with endothelial cell loss and received perforating keratoplasty (Table 2).

### 3.2. Correlation between Different Factors and Visual Acuity Outcome

Further, we tested for correlation between different factors and the visual acuity outcome after one year in order to determine possible prognostic factors. There was no significant correlation for sex (*p* = 0.79; Spearman Rho coefficient (SR): 0.038) or age (*p* = 0.76, SR: 0.035). The visual acuity at diagnosis (*p* = 0.037; SR: 0.238) and the cause of endophthalmitis (*p* = 0.002; SR: 0.34) showed a significant correlation. Patients that developed endophthalmitis after glaucoma surgery had poorer visual acuity outcomes compared to patients with endophthalmitis caused by IVI or cataract surgery (*p* = 0.048). There was no significant correlation between the occurrence of symptoms (visual deterioration: *p* = 0.69, SR: 0.045; pain: *p* = 0.92, SR: −0.011) or clinical findings (hypopyon: *p* = 0.224, SR: −0.140; conjunctival injection: *p* = 0.71, SR: −0.042; visibility of retinal vessels: *p* = 0.98, SR: −0.002; increased reflectivity of the vitreous on B-scan: *p* = 0.470, SR: 0.084). 

We performed a multivariate regression analysis, including the abovementioned factors. The BCVA at diagnosis (*p* = 0.041) remained a significant predictor, while there was no significant correlation anymore between the cause of endophthalmitis (*p* = 0.542) and the final visual acuity outcome after one year. 

### 3.3. Matched Pair Analysis between Patients with Silicone Oil Tamponade and BSS

Additionally, we identified 30 patients for a matched-pair analysis between patients with silicone oil tamponade (group 1, *n* = 15) or BSS at the end of surgery (group 2, *n* = 15) with similar baseline characteristics regarding BCVA and clinical findings (Table 3). Follow-up data were available for all 30 patients at 6–12 weeks, 28 patients at 6 months, and 26 patients at 1 year after vitrectomy.

Patients in both groups presented with a very similar BCVA (mean 1.393 logMAR vs. 1.407 logMAR) upon initial presentation (*p* = 0.967). A similar number of patients presented with vision loss (*p* = 0.624) and pain (*p* = 0.705). 

The visual outcome after vitrectomy with either silicone oil or BSS showed no significant differences at the various time points after the initial operation. About 6 to 12 weeks after vitrectomy, group 1 had a mean BCVA of 0.99 logMAR versus 0.78 logMAR in group 2 (*p* = 0.508). Six months post-vitrectomy, group 1 showed a mean BCVA of 1.17 logMAR and group 2 of 0.96 logMAR (*p* = 0.676). The mean BCVA was similar one year after vitrectomy, with a trend for better visual acuity in patients with BSS, with 1.17 logMAR in group 1 and 0.90 logMAR in group 2 (*p* = 0.684) (Table 3). 

## 4. Discussion

The treatment of postoperative endophthalmitis varies widely from center to center. There are still many unanswered questions regarding surgical management, including the choice of tamponade in patients with endophthalmitis. In this study, we evaluated a total of 82 consecutive patients treated for postoperative endophthalmitis with pars plana vitrectomy and silicone oil tamponade. 

Many studies have shown that visual acuity improves following vitrectomy in cases of endophthalmitis [19,20]. Pershing et al. analyzed the data of more than 5 million patients after cataract surgery, of whom 3.629 developed endophthalmitis (0.04%). They showed that visual acuity improved overall, but the outcome varied. Similar to these results, patients from our study showed a mean increase in BCVA from 2.1 logMAR to 1.1 logMAR after 6–12 weeks, to 1.03 logMAR after 6 months, and to 1.12 logMAR after a year following vitrectomy. Nearly 88% of all patients showed an improvement in BCVA.

The landmark EVS study is still the basis for treatment decisions for many ophthalmologists. One of the main conclusions of the EVS study was that vitrectomy was only superior to a tap and inject treatment in cases where the original BCVA was no better than light perception. However, this finding is in contrast to many recent studies that have shown that early and immediate vitrectomy may be beneficial for patients even with better visual acuity upon first presentation [11,21,22]. Although these studies are not as well designed as the original EVS study, the relatively large number of studies showing the benefits of earlier vitrectomy suggests that factors such as improved surgical devices and techniques may contribute to improved outcomes. 

In this study, we report our experience with silicone oil as a tamponade option. In our institution, the instillation of silicone oil to conclude the surgery is preferred by most surgeons. The rationale for using silicone oil is based on several factors: One is the psychological benefit for patients in terms of quicker visual rehabilitation. The second reason is the assumption that silicone oil itself may have a bacteriostatic effect and thus might lead to quieter findings at an earlier stage. As a result, it is hoped that the rate of additional interventions, such as anterior chamber washout or repeated vitrectomy, could also be lower. Moreover, silicone oil enables the surgeon to have a clearer postoperative view during funduscopy to estimate if the inflammation is indeed decreasing or if a patient would have to be revised. However, there are also some disadvantages to using silicone oil: An obvious disadvantage of its use is the need for a second surgery to remove it later on. In addition, it can be assumed that antibiotics do not mix well in silicone oil and might be excreted more quickly due to the lower distribution volume. 

There are only a few studies that analyze the outcomes after silicone oil tamponade in endophthalmitis patients. These point out that silicone oil might be beneficial in more severe cases [14,23]. Further, it might be beneficial when retinal breaks or detachments are present [17]. We had no cases of retinal detachment directly associated with endophthalmitis in our cohort. However, three patients (6.7%) developed a retinal detachment during the postoperative period and received repeated vitrectomy with silicone oil. Of these, two needed a permanent silicone oil tamponade. 

We investigated the correlation between various clinical factors and the final visual acuity outcome after one year. Patients with lower BCVA at diagnosis and endophthalmitis after glaucoma surgery tended to have poorer visual acuity outcomes after surgery. In a multivariate regression analysis, only a low BCVA at diagnosis remained a significant prognostic factor. Gower and colleagues showed that poor baseline visual acuity, advanced age at diagnosis, and more virulent organisms were important predictors of poor visual acuity outcomes [24]. A study by Lee et al. looked at different risk factors for poor visual prognosis and identified advanced age and initial visual acuity as risk factors [25]. Our results are consistent with the above studies, confirming that poor visual acuity at diagnosis seems to be a predictive risk factor for poor visual outcome after endophthalmitis. 

There are no recent studies evaluating the outcome of eyes with different tamponade options, and to the best of our knowledge, there is no study that compares silicone oil tamponade with BSS. We performed a matched-pair analysis comparing patients with silicone oil tamponade with patients with BSS at the end of surgery. Both groups had similar baseline characteristics. The development of visual acuity showed no significant differences in both groups. It has to be taken into account that the number of patients in both groups was rather low, with 15 patients per group. This is due to the fact that in our institution, silicone oil is used by most surgeons as a tamponade at the end of surgery, so we could not include more patients with complete follow-up data in group 2. Further studies on this topic are needed to evaluate the effect and provide clear recommendations on treatment guidelines. 

Only a few studies have looked at complications after vitrectomy in the management of endophthalmitis. Far et al. performed a metaanalysis and stated that the rate of new retinal detachments was 60% in a study by Agarkar et al. [26], 50% in a study by Mezad-Koursh et al. [27], and 16.67% in a study by Yannuzzi et al. [28]. Other ocular adverse events were not mentioned in the metaanalysis [29]. 

Negretti et al. performed a study with 33 patients and described that 24.2% developed a retinal detachment, 3% a macular hole, 3% a suprachoroidal hemorrhage, and 6% an enucleation/evisceration [30]. A study by Sridhar et al. described that 21.4% had a retinal detachment in their cohort, 11.4% needed more than one vitrectomy, of these, 10% received a silicone oil tamponade, and 4.3% underwent evisceration or enucleation [31]. 

In our cohort, the overall complication rate was rather low, with 17 patients (20.1%). Of these 17 patients, only a few developed major complications: three patients (6.7%) had a retinal detachment during the postoperative course and needed a repeated vitrectomy. Two patients (2.4%) had corneal decompensation and needed perforating keratoplasty. Two patients (2.4%) had prolonged inflammatory reactions after silicone oil removal. There was no case of enucleation. Overall, the rate of major complications was slightly lower in our study, indicating that silicone oil tamponade might offer some protective effect and might be beneficial with regard to postoperative complications. 

As with all retrospectively collected data, our study has some limitations since the data might be incomplete. We were not able to collect complete follow-up data for all our patients. At 6–12 weeks after vitrectomy, data on 77 patients were available in comparison to only 65 patients one year after vitrectomy. This emphasizes the need to conduct randomized, prospective studies in order to verify current results and derive sound treatment guidelines. Further, we had a rather high number of negative microbiological results (57%). Due to the retrospective nature, we were often not able to retrieve the exact reasons for that. 

Even though endophthalmitis is one of the most feared complications and treatment is essential to prevent or mitigate vision loss, there are no studies evaluating the outcome in eyes with different tamponade options. Thus, uncertainty remains in this regard since there is still a great variety in the treatment of endophthalmitis at different centers. Our results suggest that using silicone oil as a tamponade leads to beneficial results. It would be useful to conduct a randomized study comparing silicone oil with other tamponade options.

## 5. Conclusions

Our data show that early vitrectomy with silicone oil tamponade in the surgical management of postoperative endophthalmitis leads to an improvement in visual acuity, and our results are comparable to other studies using BSS or air as tamponade at the end of surgery. The choice of tamponade after performing a vitrectomy in patients with endophthalmitis should be decided individually at the discretion of the vitreoretinal surgeon. 

## Figures and Tables

**Table 1:** Patient characteristics.

	All Patients (*n* = 82)	%
**Gender**		
Male	43	52.4
Female	39	47.6
**Age (years)**		
Mean [Range, SD]	76.3 [49.1–96.2, 10.5]	
**Follow-Up Time (months)**		
Mean [Range, SD]	13.1 [0–25.1, 4.5]	
**Interval between causing event and date of onset (days)**		
Mean [Range, SD]	8.8 [1–59, 10.5]	
**Onset**		
acute (<21 days)	78	95.1
late-onset	4	4.9
**Causes of endophthalmitis**		
Intravitreal Injection	42	51.2
Cataract surgery	29	35.4
Glaucoma surgery	1	1.2
Pars-plana vitrectomy	8	9.7
IOL exchange	1	1.2
Combined vitrectomy and cataract surgery	1	1.2
**Ocular comorbidities** **(more than one possible eye)**		
None	26	31.7
Age-related macular disease	32	39.1
Diabetic Retinopathy	8	9.8
Open-Angle Glaucoma	3	3.7
Epiretinal membrane	3	3.7
Macular edema due to retinal vein occlusion	5	6.0
Uveitis	1	1.2
Neovascular glaucoma	1	1.2
Macular hole	2	2.4
Corneal disorder	1	1.2
Retinal detachment in the past	1	1.2
Irvine Gass Syndrome	2	2.4
Pseudoexfoliative Glaucoma	1	1.2
s/p perforating keratoplasty	1	1.2
**Findings at first presentation**		
**Visual deterioration**		
Yes/no	70/12	85.4/14.6
**Pain**		
Yes/no	58/24	70.7/29.3
**Hypopyon**		
Yes/no	58/24	70.7/29.3
**Conjunctival injection**		
Yes/no	65/17	79.3/20.7
**Retinal vessels visible**		
Yes/no	6/76	7.3/92.7
**Increased Reflectivity of the vitreous on B-scan**		
Yes/no	67/11	81.6/13.4
not available	4	4.9
**Pathogens**		
No result	47	57.3
*Staphylococcus epidermidis*	16	19.6
*Streptococcus agalacticae*	1	1.2
*Haemophilus influenzae*	1	1.2
Gram-positive Cocci	8	9.8
Other Staphylococci	2	2.4
*Achromobacter xylosoxidans*	1	1.2
*Staphylococcus lugdunensis*	4	4.9
*Granulicatella adiacens*	1	1.2
*Streptococcus mitis*	1	1.2

**Table 2:** Complications after vitrectomy with silicone oil.

Time Point	Complications	Number of Pat. (%)
Intraoperative	None	0 (0)
First week postoperative	Anterior chamber washout and repeated antibiotics	5 (6.1)
	Choroidal folds due to hypotony	1 (1.2)
First three months postoperative	Corneal decompensation	1 (1.2)
	Persistent fibrin and YAG-laser treatment	2 (2.4)
After three months postoperative	Retinal detachment and repeated vitrectomy	3 (6.7)
	Displacement of intraocular lens and replacement	1 (1.2)
	Corneal decompensation and perforating keratoplasty *	2 (2.4)
	Recurrence of intraocular inflammation following silicone oil removal	2 (2.4)

* One of the two patients had a repeated keratoplasty.

**Table 3:** Matched pair analysis between patients with silicone oil tamponade and BSS.

	Silicone Oil (Group 1)		BSS (Group 2)		*p* Value
	*n* = 15	%	*n* = 15	%	
**Gender**					0.464
male	8	53.3	6	40	
female	7	46.7	9	60	
**Age**	15		15		0.636
Mean [Range, SD]	75.53 [51–90, 11.8]		71.47 [49–85, 11.8]		
**Lens status**					
Phakic	0	0	0	0	
Pseudophakic	15	100.0	15	100.0	
**Interval between causing event and date of onset (days)**					0.946
Mean [Range, SD]	8.47 [1–51, 12.86]		8.07 [2–49, 12.9]		
**Onset**					0.624
acute (<21 days)	13	86.7	12	80	
late-onset	2	13.3	3	20	
**Causes of endophthalmitis**					0.381
Intravitreal Injection	7	46.7	8	53.3	
Cataract surgery	6	40.0	6	40.0	
Pars plana vitrectomy	2	13.3	0	0.0	
Combined pars plana vitrectomy and cataract surgery	0	0.0	1	6.7	
**Change in BCVA 6–12 weeks post vitrectomy**					0.591
Improved	10	76.9	9	62.9	
Worsened	3	23.1	3	23.1	
No change	0	0.0	1	7.7	
**Change in BCVA 6 months post vitrectomy**					0.574
Improved	9	75.0	8	72.7	
Worsened	3	25.0	2	18.2	
No change	0	0.0	1	9.1	
**Change in BCVA 1 year post vitrectomy**					0.260
Improved	5	83.3	6	75.0	
Worsened	1	16.7	2	25.0	
No change	0	0.0	0	0.0	
**Pathogens**					0.444
No result	9	60.0	13	86.7	
Staph. Epidermidis	3	20.0	1	6.7	
Gram-positive cocci	1	6.7	1	6.7	
*Streptococcus pneumoniae*	1	6.7	0	0	
*Staphylococcus lugdunensis*	1	6.7	0	0	

## Data Availability

The data presented in this study are available on request from the corresponding author.

## References

[1] Packer M., Chang D.F., Dewey S.H., Little B.C., Mamalis N., Oetting T.A., Talley-Rostov A., Yoo S.H. (2011). Prevention, diagnosis, and management of acute postoperative bacterial endophthalmitis. J. Cataract. Refract. Surg..

[2] Klein K.S., Walsh M.K., Hassan T.S., Halperin L.S., Castellarin A.A., Roth D., Driscoll S., Prenner J.L. (2009). Endophthalmitis after anti-VEGF injections. Ophthalmology.

[3] Haddock L.J., Ramsey D.J., Young L.H. (2014). Complications of subspecialty ophthalmic care: Endophthalmitis after intravitreal injections of anti-vascular endothelial growth factor medications. Semin. Ophthalmol..

[4] Torres-Costa S., Ramos D., Brandão E., Carneiro Â., Rosas V., Rocha-Sousa A., Falcão-Reis F., Falcão M. (2021). Incidence of endophthalmitis after intravitreal injection with and without topical antibiotic prophylaxis. Eur. J. Ophthalmol..

[5] Chen G., Tzekov R., Li W., Jiang F., Mao S., Tong Y. (2019). Incidence of Endophthalmitis after Vitrectomy: A Systematic Review and Meta-analysis. Retina.

[6] Taban M., Behrens A., Newcomb R.L., Nobe M.Y., McDonnell P.J. (2005). Incidence of acute endophthalmitis following penetrating keratoplasty: A systematic review. Arch. Ophthalmol..

[7] Endophthalmitis Vitrectomy Study Group (1995). Results of the Endophthalmitis Vitrectomy Study. A randomized trial of immediate vitrectomy and of intravenous antibiotics for the treatment of postoperative bacterial endophthalmitis. Arch. Ophthalmol..

[8] Durand M.L. (2013). Endophthalmitis. Clin. Microbiol. Infect..

[9] Tan C., Wong H., Yang F., Lee J. (2008). Outcome of 23-gauge sutureless transconjunctival vitrectomy for endophthalmitis. Eye.

[10] Damm L.J., Boden K.T., Januschowski K. (2019). Importance of vitrectomy in endophthalmitis: How immediate vitrectomy can restore visual acuity. Ophthalmologe.

[11] Januschowski K., Boden K.T., Szurman P., Stalmans P., Siegel R., Pérez Guerra N., Becker S.L., Rickmann A., Bisorca-Gassendorf L. (2021). Effectiveness of immediate vitrectomy and intravitreal antibiotics for post-injection endophthalmitis. Graefes Arch. Clin. Exp. Ophthalmol..

[12] Kitsche M., Herber R., Pillunat L.E., Terai N. (2020). Clinical and visual outcome of endophthalmitis patients: A single-center experience. Graefes Arch. Clin. Exp. Ophthalmol..

[13] Dave V.P., Joseph J., Jayabhasker P., Pappuru R.R., Pathengay A., Das T. (2019). Does ophthalmic-grade silicone oil possess antimicrobial properties?. J. Ophthalmic Inflamm. Infect..

[14] Bali E., Huyghe P., Caspers L., Libert J. (2003). Vitrectomy and silicone oil in the treatment of acute endophthalmitis. Preliminary results. Bull. De La Société Belg. D’ophtalmologie.

[15] Ozdamar A., Aras C., Ozturk R., Akin E., Karacorlu M., Ercikan C. (1999). In vitro antimicrobial activity of silicone oil against endophthalmitis-causing agents. Retina.

[16] Aras C., Ozdamar A., Karacorlu M., Ozkan S. (2001). Silicone oil in the surgical treatment of endophthalmitis associated with retinal detachment. Int. Ophthalmol..

[17] Siqueira R.C., Gil A.D.C., Canamary F., Minari M., Jorge R. (2009). Pars plana vitrectomy and silicone oil tamponade for acute endophthalmitis treatment. Arq. Bras. De Oftalmol..

[18] Azad R., Ravi K., Talwar D., Kumar N. (2003). Pars plana vitrectomy with or without silicone oil endotamponade in post-traumatic endophthalmitis. Graefe’s Arch. Clin. Exp. Ophthalmol..

[19] Pershing S., Lum F., Hsu S., Kelly S., Chiang M.F., Rich W.L., Parke D.W. (2020). Endophthalmitis after Cataract Surgery in the United States: A Report from the Intelligent Research in Sight Registry, 2013–2017. Ophthalmology.

[20] Patel S.N., Cai L.Z., Mahmoudzadeh R., Salabati M., Magan T., Obeid A., Soares R.R., Hinkle J.W., Hsu J., Dunn J.P. (2022). Endophthalmitis after Intravitreal Anti-Vascular Endothelial Factor Injections: Outcomes of Eyes Managed without Microbiologic Cultures. Am. J. Ophthalmol..

[21] Ma J., Yu Y., Zhong Y., Mao X., Fang X. (2021). Outcomes and Prognostic Factors of Posttraumatic Endophthalmitis: A Three-Year Retrospective Study. J. Ophthalmol..

[22] Sousa D.C., Jalil A., Patton N., Dhawahir-Scala F., Kim J., Charles S., Turner G., Ivanova T. (2022). Early Pars Plana Vitrectomy in Acute Endophthalmitis: The Manchester Series. Ophthalmic Surg. Lasers Imaging Retina.

[23] Kang Y.K., Shin J.P., Park H.S. (2021). Long-Term Effect of Silicone Oil Tamponade for Postoperative and Posttraumatic Bacterial Endophthalmitis. J. Ophthalmol..

[24] Gower E.W., Keay L.J., Stare D.E., Arora P., Cassard S.D., Behrens A., Tielsch J.M., Schein O.D. (2015). Characteristics of Endophthalmitis after Cataract Surgery in the United States Medicare Population. Ophthalmology.

[25] Lee J.J., Jo Y.J., Lee J.S. (2022). Clinical characteristics and risk factors for visual prognosis according to the types of infectious endophthalmitis. PLoS ONE.

[26] Agarkar S., Desai R., Jambulingam M., Sumeer S.H., Raman R. (2016). Incidence, management, and visual outcomes in pediatric endophthalmitis following cataract surgery by a single surgeon. J. Aapos.

[27] Mezad-Koursh D., Goldstein M., Heilwail G., Zayit-Soudry S., Loewenstein A., Barak A. (2010). Clinical characteristics of endophthalmitis after an injection of intravitreal antivascular endothelial growth factor. Retina.

[28] Yannuzzi N.A., Si N., Relhan N., Kuriyan A.E., Albini T.A., Berrocal A.M., Davis J.L., Smiddy W.E., Townsend J., Miller D. (2017). Endophthalmitis after Clear Corneal Cataract Surgery: Outcomes over Two Decades. Am. J. Ophthalmol..

[29] Far P.M., Yeung S.C., Farimani P.L., Qian J., Zhang A.Q., Kertes P.J., You Y., Yan P. (2021). Tap and inject versus pars plana vitrectomy for postprocedural endophthalmitis: A Meta-analysis. Retina.

[30] Negretti G.S., Chan W., Pavesio C., Muqit M.M.K. (2020). Vitrectomy for endophthalmitis: 5-year study of outcomes and complications. BMJ Open Ophthalmol..

[31] Sridhar J., Yonekawa Y., Kuriyan A.E., Joseph A., Thomas B.J., Liang M.C., Rayess N., Relhan N., Wolfe J.D., Shah C.P. (2017). Microbiologic spectrum and visual outcomes of acute-onset endophthalmitis undergoing therapeutic pars plana vitrectomy. Retina.

